# Increased face detection responses on the mooney faces test in people at clinical high risk for psychosis

**DOI:** 10.1038/s41537-021-00156-1

**Published:** 2021-05-17

**Authors:** Steven M. Silverstein, Judy L. Thompson, James M. Gold, Jason Schiffman, James A. Waltz, Trevor F. Williams, Richard E. Zinbarg, Vijay A. Mittal, Lauren M. Ellman, Gregory P. Strauss, Elaine F. Walker, Scott W. Woods, Jason A. Levin, Eren Kafadar, Joshua Kenney, Dillon Smith, Albert R. Powers, Philip R. Corlett

**Affiliations:** 1 University of Rochester Medical Center, New York, NY USA; 2 University of Maryland School of Medicine, Baltimore, MD USA; 3 Northwestern University, Evanston, IL USA; 4 Temple University, Philadelphia, PA USA; 5 University of Georgia, Athens, GA USA; 6 Emory University, Atlanta, GA USA; 7 Yale University, New Haven, CT USA; 8 Princeton University, Princeton, NJ USA; 9 Present Address: University of California, Irvine, CA USA

**Keywords:** Schizophrenia, Human behaviour

## Abstract

Identifying state-sensitive measures of perceptual and cognitive processes implicated in psychosis may allow for objective, earlier, and better monitoring of changes in mental status that are predictive of an impending psychotic episode, relative to traditional self-report-based clinical measures. To determine whether a measure of visual perception that has demonstrated sensitivity to the clinical state of schizophrenia in multiple prior studies is sensitive to features of the at-risk mental state, we examined differences between young people identified as being at clinical high risk for psychosis (CHR; *n* = 37) and non-psychiatric matched controls (*n* = 29) on the Mooney Faces Test (MFT). On each trial of the MFT, participants report whether they perceive a face in a degraded face image. The CHR group reported perceiving a greater number of faces in both upright and inverted MFT stimuli. Consistent with prior work, males reported more faces on the MFT than females in both conditions. However, the finding of greater reported face perception among CHR subjects was robustly observed in the female CHR group relative to the female control group. Among male CHR participants, greater reported face perception was related to increased perceptual abnormalities. These preliminary results are consistent with a small but growing literature suggesting that heightened perceptual sensitivity may characterize individuals at increased clinical risk for psychosis. Further studies are needed to determine the contributions of specific perceptual, cognitive, and motivational mechanisms to the findings.

## Introduction

The clinical high risk (CHR) approach to studying psychosis has led to advances in the ability to reliably identify and effectively treat those most likely to develop a psychotic disorder^1,2^. Most CHR approaches use data from clinical interviews to define at-risk subgroups and to predict conversion to a psychotic disorder; however, this approach is less than optimal in several important respects^3^. One potential way to improve clinical utility is to incorporate performance-based measures of specific neuro-computational processes that are linked to one or more aspects of schizophrenia. However, when CHR initiatives have incorporated performance-based assessments, they have often been trait-linked measures that are unlikely to be sensitive to, or predictive of, changes in mental status^4^. The incorporation of state-sensitive performance-based measures with established links to the pathophysiological mechanisms implicated in symptom genesis may allow for more objective and effective monitoring of changes in mental status that are predictive of an impending psychotic episode or a positive response to treatment^4,5^. While this approach to psychosis risk assessment is just emerging, there are already multiple studies of schizophrenia documenting that relatively brief and non-invasive behavioral and electrophysiological measures can tap into mechanisms thought to be involved in positive^6–8^, negative^9–12^, disorganized^13–15^ or motor^16^ symptoms, and that these measures are sensitive to changes in the severity level of the symptom cluster with which they are correlated^14,17,18^.

**Fig. 1:**
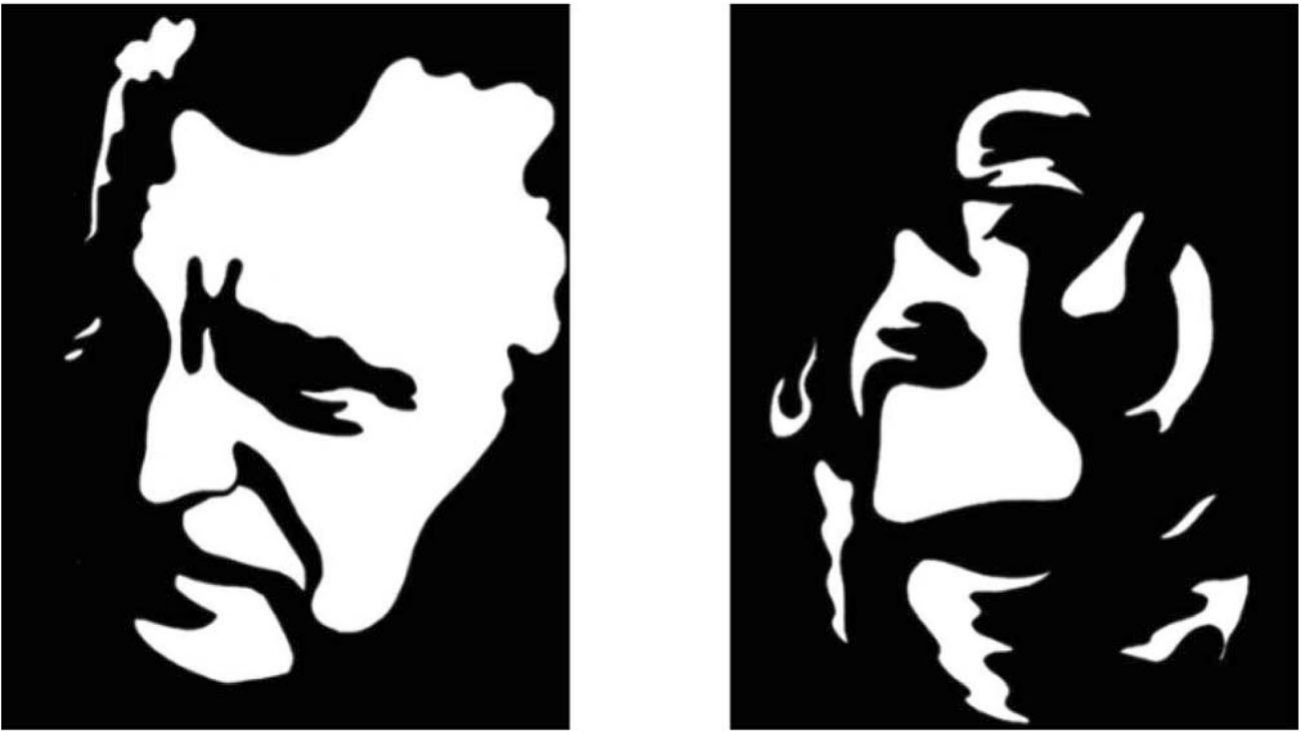
Examples of Mooney Faces Test stimuli. Left – A mid-level difficulty stimulus from the upright condition; Right – a mid-level difficulty stimulus from the inverted condition.

To inform the identification of measures that are sensitive to processes underlying symptom development and expression in the at-risk mental state, we investigated the performance of individuals at CHR on a well-validated assessment of visual perception, the Mooney Faces Test (MFT^19^). In its most basic form, participants view images of faces that have been transformed to two-tone (thresholded, or black and white only) images (Fig. 1). The images are presented one at a time, and, on each trial, subjects report whether they perceive a face in the image. Identifying faces in MFT images relies heavily on the ability to perceptually organize the black and white segments into a holistic representation (i.e., to achieve perceptual closure), culminating in face perception. The individual images are rendered such that there is a range of difficulty levels with regard to face detection across the images, from very easy to very difficult.

Because two-tone images do not lend themselves to volumetric interpretations (i.e., they are difficult to interpret as three-dimensional), perception of faces in MFT images requires the influence of top-down representations of 2D faces that are stored in memory^20^. As such, face priors applied during performance of the MFT are thought to involve a combination of stored representations of 3D (i.e., real) faces and 2D face images^21,22^. When MFT stimuli are inverted, a face is usually not perceived. This is likely for two reasons: (1) the inverted images no longer lend themselves to grouping into a face gestalt^23^ (a conclusion supported by findings of significantly less fusiform face area activation in response to inverted relative to upright MFT images)^24^; and (2) people do not have stored representations of inverted 2D face images that can be applied as templates in a top-down fashion unless they have undergone extensive training to efficiently process such images as faces.

Prior studies of the MFT in schizophrenia have reported impairments (operationalized as identification of fewer faces), consistent with the perceptual organization difficulties often observed in this population (reviewed in^25,26^). Such perceptual organization impairments are related to higher levels of disorganized symptoms in people with an established diagnosis and chronic course of schizophrenia^13^, and to reduced gamma power during task performance^27^. The relationships between these neuro-perceptual and behavioral alterations are consistent with the hypothesis that schizophrenia is characterized by widespread impairments in contextual modulation. This is defined as modifications in the grouping, timing, salience, or interpretation of stimulus-related neural signals, without changing the representation of basic stimulus features themselves. In this way, contextual modulation serves to optimize processing and adaptation across changing contexts^28^. An impairment in this canonical cortical algorithm has been hypothesized to be the basis of multiple failures of representation organization in schizophrenia (e.g., in auditory and visual perception, in thought and language)^28,29^. These data suggest that alterations in MFT performance in a CHR group would be associated with severity of disorganized symptoms. In addition, due to the top-down nature of the processing involved, and the need to evaluate the stimuli in terms of 2D and 3D face priors, as discussed above, the task may also leverage the stored knowledge and updating processes associated with predictive coding. Findings from two recent magnetoencephalography (MEG) studies are consistent with the notion that predictive processes are engaged during the MFT^30,31^. A growing literature has linked alterations in predictive processing with positive symptoms in schizophrenia^5,32–34^, suggesting that MFT performance among those at CHR for psychosis might be associated with severity of attenuated positive symptoms.

An open question was whether young people at CHR for psychosis would perceive an increased or decreased number of faces on the MFT relative to healthy controls. A decrease would be expected based on the multiple studies of reduced face perception on the MFT in participants with schizophrenia, as described above. However, there are several cases in which unmedicated first-episode patients, or high-risk subjects, evince heightened sensitivity on perceptual measures. For example, although it is well established that chronic schizophrenia is associated with impairments in the perception of low-contrast stimuli, regardless of medication status^26^, multiple studies^26^ have indicated enhanced contrast sensitivity in unmedicated first episode patients relative to controls. While no data exist on MFT performance among CHR samples, a study of perceptual organization in a sample of adult patients with schizotypal features and a history of brief, limited, intermittent psychotic symptoms (i.e., aspects of CHR status) observed enhanced performance in this high-risk group relative to controls^35^. In addition, a CHR study conducted by Teufel et al. that investigated whether prior exposure to original, unaltered images enhanced perception when viewing two-tone versions of those images (which were similar to MFT images) reported that CHR participants were superior to controls in utilizing that (experiment-provided) prior knowledge to recognize the content of the ambiguous two-tone images^36^. A second study reported in that paper demonstrated that, in a non-clinical sample of participants who were considered prone to psychosis based on measures of schizotypy, enhanced use of priors was related to psychotic-like perceptual distortions but not to quasi-delusion-like experiences. However, a study of unmedicated first episode patients using the MFT found reduced face perception^37^. Therefore, we expected that CHR participants would differ from controls in their MFT performance, and that their performance would be related to severity of perceptual abnormalities and/or disorganized symptoms specifically, but we were agnostic at the outset regarding the direction of the hypothesized significant between-group difference. Of note, unlike in the Teufel et al. study described above, we did not provide subjects with prior exposure to the unaltered face images, as we sought to determine the ‘naïve’ MFT performance of subjects, unaffected by prior exposure to any form of the images.

## Results

### Demographic characteristics

Sixty-six participants (37 CHR and 29 healthy controls (HC)) were included in this study. Thirty-five of these participants (21 CHR, 14 HC) were recruited at Northwestern University, and 31 (16 CHR, 15 HC) at the University of Georgia (see Methods). Demographic and clinical characteristics are provided in Table 1. CHR and HC groups did not differ significantly on age [*t*(64) = 1.26, *p* = 0.21], years of education [*t*(62) = −0.41, *p* = 0.68], or gender ratio [χ^2^(1) = 1.97, *p* = 0.16]. Due to the small numbers in some of the race categories, the CHR and HC groups were compared on the proportion of Caucasian vs. non-Caucasian participants. The groups did not differ significantly in racial composition using this method of classification: χ^2^ (1) = 0.93, *p* = 0.34.

**Table 1:** Demographic and clinical characteristics by group.

Characteristic	CHR (*n* = 37)	HC (*n* = 29)
Age	21.57 (2.47)	20.83 (2.25)
Gender, % female	59.5%	75.9%
Participant education (years)	14.44 (2.01)	14.64 (1.81)
Race, %		
Caucasian	56.8%	44.8%
African American	27.0%	10.4%
Asian	8.1%	34.5%
Hispanic-Latino	8.1%	3.4%
Multiracial	0.0%	6.9%
SIPS		
Positive symptom subscale	11.70 (2.98)	–
P3 Grandiose ideas	1.33 (1.45)	–
P4 Perceptual abnormalities/hallucinations	2.83 (1.25)	–
P5 Disorganized communication	1.47 (1.16)	–
Negative symptom subscale	6.73 (5.76)	–
Disorganized symptom subscale	4.41 (2.51)	–

### Between-condition differences in Mooney Faces Test performance

As expected, the frequency of reporting faces was greater for the upright condition compared to the inverted condition (73.36% vs. 33.34%, respectively): *t*(65) = 16.75, *p* < 001, *d* = 2.24.

### Between-group comparisons on Mooney Faces Test performance

**Fig. 2:**
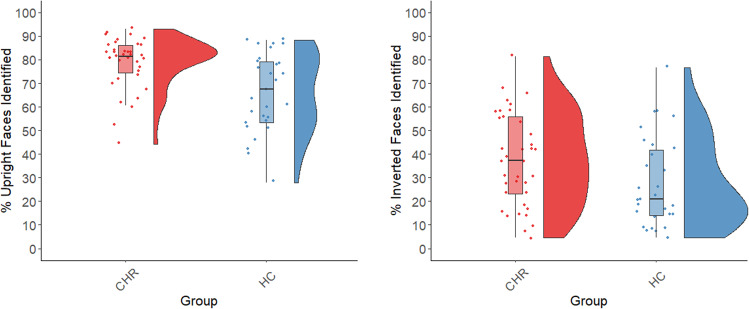
Group differences and distributions of scores (% of trials on which a face was reported) for the CHR and CON groups in the MFT conditions. Left: Box and raincloud plots showing the medians, inter-quartile ranges, and outliers for each group in the Upright Faces condition. The means were similar to the medians and were: CHR = 78.88 (SD = 10.86), CON = 66.32 (SD = 16.75). Right: Corresponding data for the Inverted Faces condition. The means were similar to the medians and were: CHR = 37.46 (SD = 19.56), CON = 28.08 (SD = 19.07).

Compared to HC participants, the CHR group reported perceiving a significantly greater number of faces in the upright condition of the MFT [*t*(64) = 3.68, *p* < 0.001, Cohen’s *d* = 0.89; see Fig. 2a]. The CHR group also reported perceiving a greater number of faces in the inverted condition, although this difference was only at a trend level [*t*(64) = 1.95, *p* = 0.055, *d* = 0.49; see Fig. 2b]. The degree to which CHR participants perceived a greater number of faces than HC participants was similar in the two conditions, as indicated by a non-significant group x condition interaction effect in a mixed-model ANOVA: *F*(1, 64) = 0.43, *p* = 0.51.

In a univariate ANOVA conducted to assess gender differences, a significant main effect was observed for gender [(*F*(1,62) = 4.82, *p* = 0.032, *d* = .57], indicating that, collapsed across groups and test conditions, males and females differed significantly on the number of faces reported. Specifically, males reported perceiving faces on 8.09% more trials than females in the upright condition, and 10.0% more trials in the inverted condition. The degree of greater face reporting among males was statistically similar across the two conditions as indicated by a non-significant gender x condition interaction term [*F*(1, 62) = 0.62, *p* = 0.44]. Post-hoc *t*-tests indicated that the difference between males and females was significant in the upright condition [*t*(64) = 2.12, *p* = .038, *d* = 0.57] and at a trend level in the inverted condition [*t*(64) = 2.00, *p* = 0.050, *d* = 0.51]. The overall pattern of findings, in terms of gender differences, was similar for the CHR and HC groups as indicated by a non-significant group × gender × condition interaction [*F*(1, 62) = 0.103, *p* = 0.79], although it must be noted that this analysis was underpowered. Female CHR subjects reported more faces than female HC subjects for upright faces [13.85% increase; *t*(42) = 3.31, *p* = 0.002, *d* = 0.998], and the difference was at a trend level for inverted faces [9.18% increase; *t*(42) = 1.68, *p* = 0.10, *d* = .51]. Among males, the difference was not significant for either the upright [6.36% increase; *t*(20) = 1.08, *p* = 0.29, *d* = 0.42] or inverted [5.16% increase; *t*(20) = 0.53, *p* = 0.60, *d* = 0.23] conditions. Although the findings were in the same direction as for females, the samples sizes in the male CHR and HC groups (*n* = 15 and 7, respectively) were smaller than the sizes for the corresponding female subgroups (*n* = 22 and 22), thereby reducing power for these subgroup analyses.

### Correlations with psychosis-risk symptoms

Among the CHR participants, there were no significant associations between the number of faces reported in either the upright or inverted condition and total scores on the SIPS positive, negative or disorganized symptom subscales, or with severity of any of the individual symptoms examined (i.e., perceptual abnormalities/hallucinations (P4), grandiosity (P3), or communication disturbance (P5); see Table 2). We also examined these correlations separately for male and female CHR participants, given the gender differences observed in task performance. Among these correlations, only one was significant: for male CHR subjects (*n* = 14), the number of faces reported in the upright condition was significantly associated with severity of perceptual abnormalities/hallucinations (*r* = 0.73, *p* = 0.003; see Table 2 and Supplemental Results for scatterplot). This correlation remained the same after Hadi outlier correction, and remained significant when calculated as a Spearman Rho (*r*_s_ = 0.66, *p* < 0.02). Nevertheless, given that this was the only significant correlation in this set of analyses, that the coefficient was unusually large, and that it was much larger for males than females (female *r* = −0.05, *p* > .99; male-female difference: *Z* = 2.59, *p* = 0.01), it is possible that this is a spurious finding rather than an indication of a link between heightened face perception on the MFT and severity of perceptual abnormalities among CHR participants. Thus, it should be viewed with caution.

**Table 2:** Bivariate correlations between SIPS symptoms and MFT performance in CHR participants.

	Full CHR sample (*n* = 37)			Male (*n* = 15)		Female (*n* = 22)	
SIPS subscale/symptom		Upright	Inverted	Upright	Inverted	Upright	Inverted
Positive symptom subscale		−0.10	0.03	0.33	0.19	−0.32	−0.07
P3 Grandiose ideas^a^		−0.17	−0.07	−0.45	−0.27	−0.21	−0.17
P4 Perceptual abnormalities^a^		0.16	0.18	0.73*	0.43	−0.05	0.12
P5 Disorganized communication^a^		0.15	0.25	0.23	0.20	0.09	0.26
Negative symptom subscale		0.29	0.28	0.46	0.43	0.28	0.28
Disorganized symptom subscale		0.18	0.20	0.47	0.11	0.08	0.34

*CHR* clinical high risk, *MFT* Mooney Faces Test, *SIPS* Structured Interview for Psychosis-Risk Syndromes.

^a^SIPS individual item scores were unavailable for 1 male CHR participant.

*p < 0.005.

## Discussion

Compared to controls, individuals at CHR reported seeing more faces on the MFT in both the upright and inverted conditions. These data imply that young people at clinical high risk for psychosis may have a heightened tendency to perceive faces in ambiguous visual stimuli that possess aspects of a facial gestalt. In a more general sense, this may reflect an increase in the signaling of social significance in situations in which information is minimal or absent. Similar to past studies, males reported perceiving a greater number of faces than females. However, the finding of greater reported face perception among CHR subjects was robustly observed among female CHR subjects relative to female controls.

This study had a number of important limitations. One is the small sample size, which is associated with low statistical power to detect small to medium effect sizes, and which could make the data unduly vulnerable to the impact of outliers, emphasizing the need for replication in a larger sample. This is the case even though we used several methods to examine and mitigate outlier effects, which we believe were minimal in this study. The sample size issue is especially relevant to the male vs. female comparisons between the CHR and control groups, as noted above. A second limitation is the lack of balance in gender composition between the CHR and HC groups. The CHR group was 41% male, and the HC group was 24% male. While this difference was not statistically significant, it is still possible that the difference could have affected group means on the MFT, given prior findings that perception of faces on the MFT may be more pronounced in males^38–40^, which is also what we observed. However, evidence that our findings are not due to a gender imbalance between groups is that although female subjects reported fewer faces than males overall, female CHR subjects reported significantly more faces in the upright condition than did HC females, and they reported more faces at a trend level in the inverted condition. A third limitation is the lack of a direct or strong indirect method for determining what subjects actually perceived, independently of what they reported perceiving. This leaves open the possibility that group differences in MFT performance could have been driven by the use of a more liberal decision criterion to indicate that a face was present among CHR participants. Unfortunately, all of our MFT stimuli contained face images (even if inverted) and thus there were no images that could truly be considered ‘noise’ stimuli, precluding a signal detection analysis to distinguish between perceptual and decisional contributions to the task. See Supplemental Discussion for a further discussion of issues involved in using signal detection theory, or generating psychometric functions based on stimulus difficulty level, using the original MFT stimulus set. In future studies, it would be possible to perform a signal detection analysis if either scrambled faces, or, preferably, ‘Mooney objects’ (which contain no face components) were used as catch stimuli. There are also newer versions of the MFT that have validated difficulty scores for a large number of items^41^, which would facilitate the generation of psychometric functions across stimuli for subjects and groups. For the stimulus set used in the present study, no such data are available (see Supplemental Discussion for more on this issue).

The contributions of perceptual changes versus adoption of a more liberal criterion for responding that a face was seen could also be addressed by including additional psychophysiological or behavioral measures. For example, one could investigate fusiform face area (FFA) activation in response to upright vs. inverted Mooney face images. Previous fMRI studies indicate that activation of the FFA is significantly greater in response to upright relative to inverted^20,24^ or scrambled^42^ faces. Similar findings have been observed using MEG^27^. Therefore, groups could be compared on the extent to which their reports of seeing faces correspond to FFA activation. An EEG study demonstrated that perception of upright faces was associated with a significant increase in synchronized gamma-band activity between parietal-occipital and fronto-temporal regions, and that this increase was not observed during viewing of inverted versions of the same images^43^. Thus, differences in level of synchrony increase in response to MFT images may be helpful in classifying instances of face perception vs. reports driven by post-perceptual (decisional) factors. Future studies could also ask subjects to report what kind of face they perceive (e.g., young, old, male, female), and then these responses could be compared to the characteristics of the person depicted in the original, unaltered, image^13^.

In addition to generating more precise estimates of perceptual versus decisional contributions to MFT performance across groups, the contribution of other impairments needs to be taken into account. For example, there is evidence that attention to a stimulus induces a conservative perceptual bias^44^ and a shift towards stronger evaluation of sensory evidence (and therefore reduced reliance on priors)^45^; such findings raise the possibility that some of the effects we observed could have been due, in part, to less focused attention and subsequently a more liberal and prior-based task strategy among CHR subjects.

While the present data do not allow for strong conclusions to be drawn regarding the causes of the increase in reported face perception in CHR subjects, the findings are intriguing, and they suggest several specific directions for larger follow-up studies. Primary among these, the significantly greater number of reported faces in the CHR group raises the possibility of heightened perceptual sensitivity during the prodromal period of psychosis. As noted above, this has been observed in perceptual organization in high-risk subjects (whereas first episode and later episode schizophrenia patients demonstrate impairments), and in contrast sensitivity in unmedicated first-episode patients (whereas chronically ill patients show an impairment). The significant relationship between greater face reporting and perceptual abnormalities in male CHR subjects is consistent with the data of Teufel et al. and with the hypothesis that perceptual changes and an excessive reliance on priors (i.e., alterations in predictive processing) may be related characteristics of CHR patients with attenuated psychotic symptoms. This hypothesis was also suggested in a recent paper in which face detection in a task with 40 face-in-noise stimuli and 60 pure noise stimuli was examined in a group of 39 young adults^46^. Results indicated that increased reporting of faces in response to noise stimuli was correlated with scores on the Cardiff Anomalous Perception Scale^47^ (*r*_s_ = .50, *p* = 0.001) and scores on the Peters Delusion Inventory^48^ (*r*_s_ = 0.44, *p* = 0.005); the authors suggested that these findings may reflect the influence of overly strong priors for socially meaningful stimuli in ambiguous contexts in people prone to psychosis. In the present study, however, the general lack of strong correlations between MFT scores and symptoms among the CHR participants suggests that it is best to remain agnostic as to MFT-symptom relationships, and the potential for shared mechanisms, until further data are available. The major interpretive issue involving the study, that of perceptual versus decisional contributions to the data, must be considered an open question. Given the unexpectedly (in the context of literature on schizophrenia and CHR groups in general) ‘superior’ performance of the CHR group, however, further exploration of this issue could be potentially fruitful for risk detection initiatives, especially to the extent that the findings are found to have a perceptual rather than a decisional or motivational basis.

## Methods

### Participants

CHR subjects were recruited from specialized CHR clinics at two sites: The Adolescent Development and Preventative Treatment (ADAPT) Program at Northwestern University (PI: Mittal), and the Georgia Psychiatric Risk Evaluation Program (G-PREP) at the University of Georgia (PI: Strauss). Both programs received referrals from local clinicians (e.g., psychiatrists, psychologists, social workers, school psychologists) to perform diagnostic assessment and monitoring evaluations for youth reporting experiencing psychotic-like symptoms, and for youth with a family history of a psychotic disorder who are reporting an increase in general psychopathology symptoms (e.g., depression, anxiety). All CHR participants met criteria for a psychosis-risk syndrome according to the Structured Interview for Psychosis-Risk Syndromes (SIPS)^49,50^. Healthy control (HC) participants were recruited from surrounding communities via online and printed advertisements. Controls did not meet lifetime diagnostic criteria for a DSM-5 psychiatric disorder as determined by the SCID-5^51^, had no family history of psychosis, no lifetime history of neurological disorders, and were not currently prescribed psychotropic medications.

The study was approved by the internal review boards (IRBs) at Northwestern University and the University of Georgia. All participants provided written informed consent, on IRB-approved consent forms, for their involvement in the study.

### Mooney faces test

In the original version of the test^19^, there were 50 degraded (two-tone) black and white images of faces of men, women, and children of various ages. We used 43 of the 44 images in Landsell’s^52^ adaptation of the test. Each image was presented twice, once in an upright orientation and once in an inverted orientation, in a pseudorandom order. It was expected that participants would report perceiving faces in most of the upright images, and in few of the inverted images (consistent with prior studies). The task instructions were simple and presented on a single screen: “Welcome to the experiment! At the beginning of each trial you will see a black and white image. If you believe the image is a face, please press the 1 key on the keyboard. If you do not believe the image is a face, please press the 0 key.” The test was implemented in PsychoPy 1.90.1^53,54^ using Python 2.

### Symptom assessment

Symptoms were assessed using the SIPS^49,50^. To minimize the number of correlational analyses that were undertaken, we limited our examination of MFT-symptom relationships to only the following symptoms: (1) perceptual abnormalities/hallucinations (SIPS item P4), as we anticipated that this positive symptom would be most related to MFT performance if the tendency to report perceiving faces was related to altered predictive coding; (2) grandiose ideas (P3), a positive symptom we expected to be independent of MFT performance, based on^36^; and (3) disorganized communication (P5), given that severity of disorganization has been found to be related to perceptual organization impairment^13,15,55^. We also examined correlations with total scores on the SIPS positive, negative, and disorganized symptom subscales. We predicted that none of the correlations with subscale scores would be statistically significant, given the inclusion of multiple items which we predicted would be weakly related to predictive coding (e.g., grandiosity) and impaired contextual modulation (e.g., impairment in personal hygiene) on the positive and disorganized subscales respectively, and prior findings suggesting that the mechanisms involved in negative symptoms and MFT performance do not overlap.

### Data analysis

The groups were compared using independent-samples t-tests and mixed-model analyses of variance. Group differences in gender and race were examined using a Pearson chi-square test. Gender differences and the group x gender interaction were examined using ANOVAs. These were explored because in non-clinical samples, it has been observed that males tend to report perceiving more faces than females on the MFT^38–40^. Pearson correlations were calculated both with and without the Hadi correction^56,57^ to exclude the influence of outlying values. When *r* values were similar in both cases, Pearson *r* values are reported. When the values differed, Spearman’s Rho (*r*_s_) values were calculated and are reported. In one case, both Pearson and Spearman values are reported for the sake of comparison. All p values are two-sided.

### Reporting summary

Further information on research design is available in the Nature Research Reporting Summary linked to this article.

## Supplementary information

Supplementary Information

Reporting Summary

## Data Availability

The data from this study are available from the corresponding author upon reasonable request.

## Code availability

The code used in this study, to present the Mooney Faces Test (in PsychoPy) and to score the individual subject raw data files (in Visual Basic for Applications), are available from the corresponding author upon reasonable request.
