# Supplementary material for: Increased face detection responses on the mooney faces test in people at clinical high risk for psychosis
Source: NPJ Schizophr. 2021 May 17;7:26. doi: 10.1038/s41537-021-00156-1 (PMC8129098; doi:10.1038/s41537-021-00156-1)
Supplement: Supplementary file 1 — Supplementary Information [file 41537_2021_156_MOESM1_ESM.pdf]

Supplementary Results

Supplementary Figure 1.

Scatterplots showing relationships between SIPS Perceptual Abnormalities score (P4) and percent of face-positive responses in the upright (Left panel) and inverted (Right panel) conditions of the Mooney Faces Task for male CHR subjects (n=14 with available data). Data points are surrounded by a convex hull in both panels. Upright condition: Pearson $r = .73$, $p = .003$, $p<.02$). Inverted condition: Pearson $r = .43$, $p = .37$.

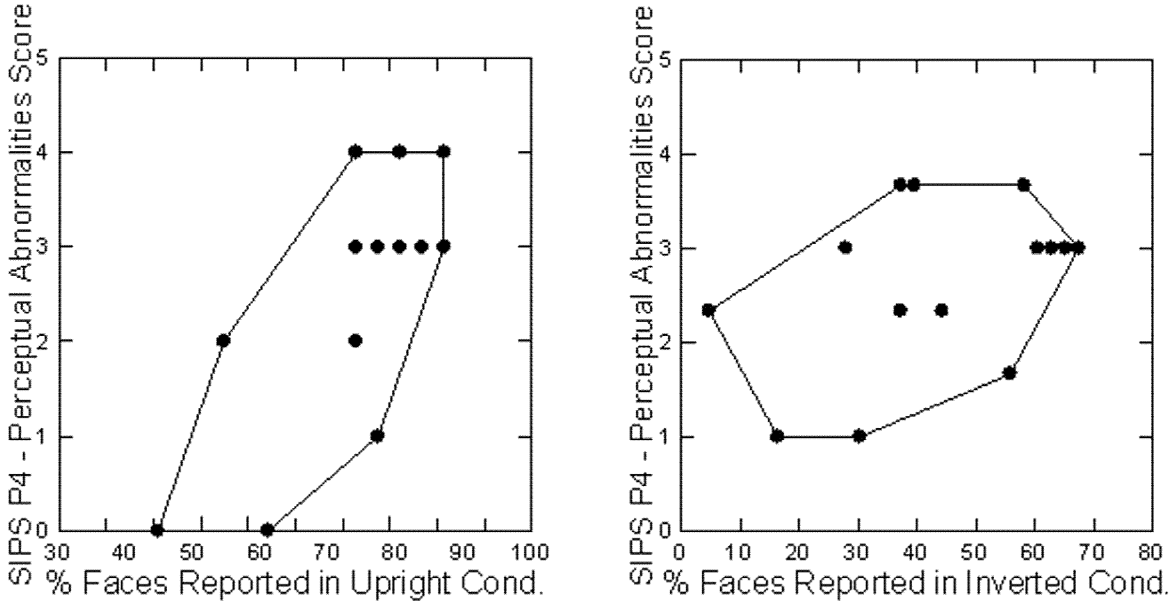

Supplementary Discussion

We replicated the previous observation that males reported more faces than females on the MFT[1-3], but the explanation of these effects is not clear. Despite many studies of sex differences in cognition, there is little research on such differences on visual perceptual tasks[4]. One study of perceptual organization in schizophrenia patients found superior performance in females[5], and another (with a higher functioning sample) found no sex differences[6]. While it has been proposed that visual region volumes (which are generally larger in males, as is brain size overall) are related to better abilities in a number of visual processing domains[7], literature on face perception indicates that fusiform gyrus volumes are *inversely* related to face detection[8], and

that males and females do not differ in volumes of this region[9]. In short, to our knowledge, there is no clear indication in the literature at this point that can explain an increased reporting of faces among males on the MFT.

Regarding the issue of the extent to which inverted MFT stimuli can be considered to be noise stimuli, although faces are typically reported less often in the inverted condition, they are still reported for approximately 25% of the stimuli in this condition on average[10-12], likely due to the perception of face components (e.g., eyes, mouth) that look relatively similar in both orientations. In Mooney's original paper on the MFT, 44% of the inverted images were recognized as faces by a subset of participants (range = 11% to 89% of subjects per inverted stimulus).

Regarding the possibility of generating a psychometric function based on difficulty ratings of MFT stimuli, while a rank ordering of difficulty can be constructed using responses of 30 adults in the original 1957 paper by Mooney[13], we did not consider this a firm basis for deriving an intra-item-focused sensitivity metric. This is because those rankings corresponded to degree of difficulty of correctly categorizing the images into one of six categories (e.g., boy, grown woman, old man) and not to degree of difficulty detecting a face. In addition, a later study[14] revised Mooney's interpretation of correct vs incorrect answers based on an item analysis of a larger group of subjects, but the specific item ratings were never reported. Thus, Mooney's original rankings should be not be considered a strong basis for attempting to reconstruct detection (i.e., present/absent) difficulty rankings. Of note, in a newly developed set of MFT stimuli, performance was not affected by the proportion of scrambled face trials included in the stimulus set, when this proportion was systematically manipulated[15] . This suggests that performance on the traditional MFT is not affected by overall absolute or perceived number of

face stimuli, and argues against a response bias interpretation in our data (i.e., one in which one group of subjects was more influenced by responses on prior trials than the other group). However, this possibility needs to be tested directly via inclusion of noise stimuli in a between-groups design. It is also possible that some subjects who could not perceive a face could nevertheless detect facial features such as eyes or a mouth (which look relatively similar in both upright and inverted face images), and based their responses on detection of these features alone. However, there is no *a priori* reason to believe this would occur more for CHR subjects than controls, or more among males than females. In addition, such a strategy would be expected to arise in cases of impaired perceptual organization, but as noted in the Introduction, the only study of perceptual organization in people at clinical high risk for psychosis found enhanced perceptual organization abilities.

Supplementary References

1 Foreman, N. Correlates of performance on the Gollin and Mooney tests of visual closure. *J Gen Psychol* **118**, 13-20, doi:10.1080/00221309.1991.9711129 (1991).

2 Verhallen, R. J. *et al.* An online version of the Mooney Face Test: phenotypic and genetic associations. *Neuropsychologia* **63**, 19-25, doi:10.1016/j.neuropsychologia.2014.08.011 (2014).

3 Vigen MP, G. R., Embree LJ. Adults' performance on a measureof visual closure. *Perceptual and motor skills* **55**, 943-952, doi:2. http://dx.doi.org/10.2466/pms.1982.55.3.943 (1982).

4 Shaqiri, A. *et al.* Sex-related differences in vision are heterogeneous. *Scientific reports* **8**, 7521, doi:10.1038/s41598-018-25298-8 (2018).

5 Joseph, J., Bae, G. & Silverstein, S. M. Sex, symptom, and premorbid social functioning associated with perceptual organization dysfunction in schizophrenia. *Front Psychol* **4**, 547, doi:10.3389/fpsyg.2013.00547 (2013).

6 Strauss, M. E. *et al.* Temporal Stability and Moderating Effects of Age and Sex on CNTRaCS Task Performance. *Schizophr Bull* **40**, 835-844, doi:10.1093/schbul/sbt089 (2014).

7 Vanston, J. E. & Strother, L. Sex differences in the human visual system. *J Neurosci Res* **95**, 617-625, doi:10.1002/jnr.23895 (2017).

8 McGugin, R. W., Van Gulick, A. E. & Gauthier, I. Cortical Thickness in Fusiform Face Area Predicts Face and Object Recognition Performance. *J Cogn Neurosci* **28**, 282-294, doi:10.1162/jocn_a_00891 (2016).

9 Shah, M., Kurth, F. & Luders, E. The impact of aging on the subregions of the fusiform gyrus in healthy older adults. *J Neurosci Res* **99**, 263-270, doi:10.1002/jnr.24586 (2021).

10 Andrews, T. J. & Schluppeck, D. Neural responses to Mooney images reveal a modular representation of faces in human visual cortex. *Neuroimage* **21**, 91-98, doi:10.1016/j.neuroimage.2003.08.023 (2004).

11 George, N. *et al.* Contrast polarity and face recognition in the human fusiform gyrus. *Nat Neurosci* **2**, 574-580, doi:10.1038/9230 (1999).

12 Tong, F., Nakayama, K., Vaughan, J. T. & Kanwisher, N. Binocular rivalry and visual awareness in human extrastriate cortex. *Neuron* **21**, 753-759, doi:10.1016/s0896-6273(00)80592-9 (1998).

13 Mooney, C. M. Age in the development of closure ability in children. *Canadian Journal of Psychology* **11**, 216-226 (1957).

14 Landsell, H. Effect of extent of temporal lobe ablations on two lateralized deficits. *Journal of Physiology and Behavior* **3**, 271-273 (1968).

15 Schwiedrzik, C. M., Melloni, L. & Schurger, A. Mooney face stimuli for visual perception research. *PLoS One* **13**, e0200106, doi:10.1371/journal.pone.0200106 (2018).
