# Supplementary material for: Increased face detection responses on the mooney faces test in people at clinical high risk for psychosis
Source: NPJ Schizophr. 2021 May 17;7:26. doi: 10.1038/s41537-021-00156-1 (PMC8129098; doi:10.1038/s41537-021-00156-1)
Supplement: Supplementary file 2 — Reporting Summary [file 41537_2021_156_MOESM2_ESM.pdf]

nature research

Corresponding author(s): Steven M. Silverstein

Last updated by author(s): Feb 17, 2021

Reporting Summary

Nature Research wishes to improve the reproducibility of the work that we publish. This form provides structure for consistency and transparency in reporting. For further information on Nature Research policies, see our Editorial Policies and the Editorial Policy Checklist.

Statistics

For all statistical analyses, confirm that the following items are present in the figure legend, table legend, main text, or Methods section.

n/a	Confirmed	
☐	☒	The exact sample size (*n*) for each experimental group/condition, given as a discrete number and unit of measurement
☐	☒	A statement on whether measurements were taken from distinct samples or whether the same sample was measured repeatedly
☐	☒	The statistical test(s) used AND whether they are one- or two-sided<br>*Only common tests should be described solely by name; describe more complex techniques in the Methods section.*
☒	☐	A description of all covariates tested
☐	☐	A description of any assumptions or corrections, such as tests of normality and adjustment for multiple comparisons
☐	☒	A full description of the statistical parameters including central tendency (e.g. means) or other basic estimates (e.g. regression coefficient) AND variation (e.g. standard deviation) or associated estimates of uncertainty (e.g. confidence intervals)
☐	☒	For null hypothesis testing, the test statistic (e.g. *F*, *t*, *r*) with confidence intervals, effect sizes, degrees of freedom and *P* value noted<br>*Give P values as exact values whenever suitable.*
☒	☐	For Bayesian analysis, information on the choice of priors and Markov chain Monte Carlo settings
☒	☐	For hierarchical and complex designs, identification of the appropriate level for tests and full reporting of outcomes
☐	☒	Estimates of effect sizes (e.g. Cohen's *d*, Pearson's *r*), indicating how they were calculated

Our web collection on statistics for biologists contains articles on many of the points above.

Software and code

Policy information about availability of computer code

Data collection	The Mooney Faces Test was programmed in PsychoPy version 3.0. Code to score the individual subject output files was implemented in Microsoft Visual Basic for Applications version 7.1.
Data analysis	Data were analyzed using SYSTAT version 13.

For manuscripts utilizing custom algorithms or software that are central to the research but not yet described in published literature, software must be made available to editors and reviewers. We strongly encourage code deposition in a community repository (e.g. GitHub). See the Nature Research guidelines for submitting code & software for further information.

Data

Policy information about availability of data

All manuscripts must include a data availability statement. This statement should provide the following information, where applicable:

- Accession codes, unique identifiers, or web links for publicly available datasets
- A list of figures that have associated raw data
- A description of any restrictions on data availability

The data that support the findings of this study are available from the corresponding author upon reasonable request.

Field-specific reporting

Behavioural & social sciences study design

All studies must disclose on these points even when the disclosure is negative.

Study description	Quasi-experimental design. Quantitative data. Comparison of people at high risk for psychosis with a matched healthy control group on a test of visual perception.
Research sample	Young adults seeking treatment at a clinic for people at high risk for psychosis, and a matched control group.
Sampling strategy	Convenience samples.
Data collection	Computerized visual perception test, clinical symptom interviews.
Timing	Spring of 2019.
Data exclusions	None.
Non-participation	No participants dropped out. All data were collected in a single session.
Randomization	Non-random assignment, based on whether a person was a patient at the high risk clinic, or a control participant from the community.

Reporting for specific materials, systems and methods

We require information from authors about some types of materials, experimental systems and methods used in many studies. Here, indicate whether each material, system or method listed is relevant to your study. If you are not sure if a list item applies to your research, read the appropriate section before selecting a response.

Materials & experimental systems

n/a	Involved in the study	
☒	☐	Antibodies
☒	☐	Eukaryotic cell lines
☒	☐	Palaeontology and archaeology
☒	☐	Animals and other organisms
☐	☒	Human research participants
☐	☒	Clinical data
☒	☐	Dual use research of concern

Methods

n/a	Involved in the study	
☒	☐	ChIP-seq
☒	☐	Flow cytometry
☒	☐	MRI-based neuroimaging

Human research participants

Policy information about studies involving human research participants

Population characteristics	Men and women, age 16-30, no restrictions on race or ethnicity.
Recruitment	Flyers at local clinics (for patients) or in community settings (for control subjects).
Ethics oversight	University of Georgia and Northwestern University IRBs.

Note that full information on the approval of the study protocol must also be provided in the manuscript.

Clinical data

Policy information about clinical studies

All manuscripts should comply with the ICMJEguidelines for publication of clinical research and a completedCONSORT checklist must be included with all submissions.

Clinical trial registration	NA
Study protocol	NA, this was an unfunded pilot study.
Data collection	Data collected at the clinical high risk programs at the University of Georgia and at Northwestern University, in 2019.
Outcomes	We used a well-validated perceptual test as our primary dependent variable.
